# Interleukin-1β and tumor necrosis factor-α: reliable targets for protective therapies in Parkinson’s Disease?

**DOI:** 10.3389/fncel.2013.00053

**Published:** 2013-04-29

**Authors:** María C. Leal, Juan C. Casabona, Mariana Puntel, Fernando J. Pitossi

**Affiliations:** Institute Leloir Fundation - IIBBA-CONICETBuenos Aires, Argentina

**Keywords:** interleukin-1β, tumor necrosis factor-α, Parkinson’s Disease, neurodegeneration, inflammation

## Abstract

Neuroinflammation has received increased attention as a target for putative neuroprotective therapies in Parkinson’s Disease (PD). Two prototypic pro-inflammatory cytokines interleukin-1β (IL-1) and tumor necrosis factor-α (TNF) have been implicated as main effectors of the functional consequences of neuroinflammation on neurodegeneration in PD models. In this review, we describe that the functional interaction between these cytokines in the brain differs from the periphery (e.g., their expression is not induced by each other) and present data showing predominantly a toxic effect of these cytokines when expressed at high doses and for a sustained period of time in the *substantia nigra pars compacta* (SN). In addition, we highlight opposite evidence showing protective effects of these two main cytokines when conditions of duration, amount of expression or state of activation of the target or neighboring cells are changed. Furthermore, we discuss these results in the frame of previous disappointing results from anti-TNF-α clinical trials against Multiple Sclerosis, another neurodegenerative disease with a clear neuroinflammatory component. In conclusion, we hypothesize that the available evidence suggests that the duration and dose of IL-1β or TNF-α expression is crucial to predict their functional effect on the SN. Since these parameters are not amenable for measurement in the SN of PD patients, we call for an in-depth analysis to identify downstream mediators that could be common to the toxic (and not the protective) effects of these cytokines in the SN. This strategy could spare the possible neuroprotective effect of these cytokines operative in the patient at the time of treatment, increasing the probability of efficacy in a clinical setting. Alternatively, receptor-specific agonists or antagonists could also provide a way to circumvent undesired effects of general anti-inflammatory or specific anti-IL-1β or TNF-α therapies against PD.

## PARKINSON’S DISEASE AND NEUROINFLAMMATION

Parkinson’s Disease (PD) is a neurodegenerative disorder affecting over 1% of the population above 65 years of age (Lesage and Brice, 2009; Obeso et al., 2010; Coelho and Ferreira, 2012). Its cardinal symptoms are bradykinesia (slowness of movement), tremor, rigidity, and postural imbalance (Lesage and Brice, 2009; Obeso et al., 2010; Coelho and Ferreira, 2012). Gait disturbances, swallowing and speech problems, autonomic dysfunctions, and cognitive decline can also be present in PD patients (Obeso et al., 2010). Dopaminergic neurons in the substantia nigra pars compacta (SN) projecting to the striatum are the main, but not the only neurons degenerating in PD. The death of these dopaminergic neurons causes the classical motor symptoms of these patients. When patients suffer motor symptoms, over 60% of all dopamine neurons within specific regions of the basal ganglia may have been lost (Bernheimer et al., 1973). Current therapies are focused on the restoration of dopamine levels in the striatum by the administration of dopamine precursors, dopamine agonists, or selective inhibitors of dopamine uptake, among other pharmacological strategies (Obeso et al., 2010). Another therapeutic approach is the implantation of electrodes to reduce the overexcitation of specific brain areas such as the subthalamic nucleus (Okun, 2012). However, no therapy is available that can slow down or halt the progression of PD or regenerate the affected brain regions. The lack of improved therapies is attributable to the incomplete knowledge available on the etiology and physiopathology of the disease. The delayed diagnosis of PD also adds difficulty in designing new therapeutic avenues.

Although a load of valuable information has been obtained during the last years, the etiology of not more than 5% of PD cases has been revealed to be caused by genetic defects (Smith et al., 2012). Environmental factors such as pesticides have also been shown to trigger idiopathic PD (Wirdefeldt et al., 2011). In addition, impairment of genes involved in detoxification can contribute to the risk to develop PD, as some polymorphisms and mutations that increase or reduce the activity of the paraoxonase 1 gene could be reducing or increasing the risk for PD, respectively (Benmoyal-Segal et al., 2005; Belin et al., 2012).

In parallel to an intense effort to unravel the etiology of PD, increasing evidence is now available on the physiopathology of this illness. It is foreseen that the understanding of the pathological process of neurodegeneration and the subsequent identification of the molecules mediating this process will allow the detection of new therapeutic targets. These novel targets should enable the design of disease-modifying therapies, slowing down or halting disease progression. The involvement of neuroinflammation in the pathogenesis of PD in patients is supported by several reports that anti-inflammatory medication use is associated with a reduction in the risk of PD (Chen et al., 2005). Moreover, inflammation biomarkers, like high-sensitivity C-reactive protein and interleukin-6 (IL-6) were found upregulated in human patients (Chen et al., 2008; Soreq et al., 2008; Song et al., 2011).

An ubiquitous physiopathological feature of the degenerating SN in animal models and PD patients is the presence of robust microglial activation in that brain region. Not so long ago, microglial activation has been regarded as a physiological consequence of neuronal death, whose function was solely to remove neuronal debris. This vision was wrong. Microglial activation can lead to toxic but also protective effects on the on-going neurodegenerating neurons of the SN (reviewed in Roca et al., 2011). This review will be focused on the functional consequences of two prototypical microglial-derived cytokines [IL-1β and TNF-α] on PD physiopathology.

## PD AND MICROGLIAL ACTIVATION

Microglial cells are the resident macrophages of the brain (Perry et al., 2010). They are main mediators of the innate immune response and account for ca. 10% of all brain cells (Perry et al., 2010). Upon changes in their surrounding environment, microglial cells become activated, changing their morphology and secreting a variety of molecules with functional consequences in the central nervous system (CNS). The activation of microglial cells in the degenerating SN in PD is ubiquitous. It has been found in mice, rats, and monkeys models of PD based on neurotoxins such as 6-hydroxydopamine (6-OHDA), 1-methyl-4-phenyl-1,2,3,6-tetrahydropyridine (MPTP) pesticides such as rotenone or alpha-synuclein overexpression (reviewed in Roca et al., 2011). In addition, robust microglial activation has been found in the SN in post-mortem samples and by imaging techniques in PD patients (reviewed in Roca et al., 2011).

Until recently, microglial activation was associated with a pro-inflammatory environment. Our group has shown that microglial activation during neurodegeneration in the SN leads to pro-inflammatory cytokine transcription but not translation in these cells (Depino et al., 2003). This “atypical” activation of microglial cells, associated with neurodegeneration but not with inflammation was in line with previous observations in prion and Alzheimer’s disease (AD; Perry et al., 2002). From these works, it became clear that microglial activation does not always lead to an inflammatory response. On the other hand, an inflammatory response always involves microglial activation. Therefore, microglial activation is a necessary but not a sufficient feature of neuroinflammation.

As stated above, activated microglia will secrete a plethora of molecules with functional consequences for their environment in general, and the dying neurons in particular. It is important to highlight that the functional consequences of microglial activation will depend on the array of molecules they secrete. This review will focus on the functional role on the degenerating SN of two prototypic, microglial secreted pro-inflammatory cytokines, IL-1β and TNF-α. These two cytokines are potent mediators of microglial function and key to modulate the complex network of interactions of microglial-secreted molecules. Provided that microglial activation is ubiquitous and that these two cytokine exert such a potent influence on the final functional outcome of microglial activation, they have been proposed as possible targets to be used in neuroprotective, disease-modifying therapies against PD. In the following review, we will discuss the role of these molecules on neuronal death in the SN, their interaction (which has distinct features in the brain compared with the periphery) and the parameters that will affect their final effect on the dopaminergic neurons.

Most of the available evidence leads to conclude that IL-1β and TNF-α are toxic for the dopaminergic neurons of the SN. However, completely opposite results have also been obtained, showing IL-1β and TNF-α-derived neuroprotective effects. Several groups including ours, have found that the final effect of these cytokines on dopaminergic cell viability depends on many variables such as concentration, duration of expression and state of activation of the target or neighboring cells (Depino et al., 2003; Ferrari et al., 2004, 2006).

Before presenting and discussing the data that lead to these conclusions, a general overview of each cytokine is presented, necessary for the full understanding of the biological effects of these cytokines on neurodegeneration.

## IL-1β – GENERALITIES

IL-1β was first described by around 1943 as an agent coming from rabbit peritoneal cells. Its first biological property to be discovered was to produce fever, and therefore was termed endogenous pyrogen (Dinarello, 1996). IL-1β is a pro-inflammatory cytokine that participates in several neuroimmunological and neurophysiological activities in the CNS. IL-1 family comprises eleven members, among which IL-1α and IL-1β are the best studied. Both proteins have a 25% of sequence similarity at the protein level despite being products of different genes (Allan et al., 2005). They are produced as large precursor protein by many cellular types on peripheral and central immune system, such as blood monocytes and tissue macrophages (Schumann et al., 1998; Denes et al., 2012). Pro-IL-1β (31- to 33-kDa) is biologically inactive and then proteolytically processed by caspase-1 resulting in a functional secretory 17-kDa protein (Rubartelli et al., 1990; Schumann et al., 1998).

There are multiple steps of regulation of IL-1β production and activity, including transcription, translation, cleavage, and cellular secretion (Allan et al., 2005). In macrophages for example, IL-1β activation involves two distinct, sequential and intracellular signals to produce IL-1β transcription and translation (van de Veerdonk et al., 2011). Soluble IL-1β as well as IL-1α induces cellular response through its binding to transmembrane receptors: IL-1R1 or IL-1R3. A third specific ligand, the IL-1 receptor antagonist (IL-1ra) binds the IL-1R1 with similar specificity and affinity but does not activate the receptor and trigger downstream signaling (Weber et al., 2010). IL-1R1 is found ubiquitously and IL-1R3 presents preferential neural tissue expression (Qian et al., 2012). This is followed by recruitment of an accessory protein (IL-1RAcP, called co-receptor) and then this trimeric complex starts signaling by assembling the adaptor protein MyD88 to the Toll-IL-1R1 (TIR) domain. Recently, a novel isoform of co-receptor (IL-1RAcPb) was described, expressed mainly in the CNS (Huang et al., 2011). An additional receptor, IL-1R2, binds IL-1 with high affinity but does not transduce IL-1 signaling since it lacks an intracellular domain (Dinarello, 2011). Several kinases participate in the canonical cascade including members of IL-1 receptor-activated protein kinase (IRAK) family (Gosu et al., 2012) that ends in the nuclear translocation of the transcription factor NF-κB (nuclear factor-kappaB). Other signaling pathways activated by IL-1β involve mitogen-activated protein family (MAP) and Src kinases (Lu et al., 2005; Huang et al., 2011). In the healthy brain, the levels of IL-1β are very low but its levels are increased after several conditions, including damage and peripheral inflammation (Pitossi et al., 1997; Laye et al., 2000). Induced central IL-1β is mainly produced by microglia (Herx et al., 2000) but also by neurons (Silverman et al., 2005). In the rat brain IL-1R1 mRNA is mainly localized in neurons, in the hypothalamus and hippocampus (Turnbull and Rivier, 1999).

Neurons have been shown to be particularly susceptible to IL-1β-mediated toxicity. For example, Purkinje neurons suffer apoptosis when microglia releases IL-1β and TNF-α (Kaur et al., 2012). IL-1β primes neurons to undergo excitotoxic death. IL-1β recruits various members of the MAP kinase pathway that are known to control neurodegeneration mediated by over-stimulation of the glutamate receptor (Doble, 1999). For example, it has been demonstrated that during an acute attack of Multiple Sclerosis (MS), inflammation increases brain IL-1β signaling, which enhances in turn glutamate-mediated synaptic excitability (Rossi et al., 2012).

## FUNCTIONAL ROLE OF IL-1β IN PARKINSON’S DISEASE

IL-1β is a pro-inflammatory cytokine with pleiotropic biological actions in the periphery and the brain. IL-1β has been found in the cerebrospinal fluid and post-mortem striata of PD patients (Mogi et al., 1994, 1996; Nagatsu et al., 2000). In addition, IL-1β gene polymorphisms have been associated with age-at-onset of sporadic PD (Nishimura et al., 2000; McGeer et al., 2002; Wahner et al., 2007), although not all reports lead to similar conclusions (Nishimura et al., 2000; Liu et al., 2010; Pascale et al., 2011; Chu et al., 2012).

As stated above, the final functional outcome of IL-1β on dopaminergic cell loss will depend on variables such as concentration, duration of expression, and timing among stimuli. In terms of the influence of the duration and levels of IL-1β expression, it is irrefutable that the sustained expression of IL-1β in the SN at pro-inflammatory levels causes irreversible and pronounced dopaminergic neuronal loss in the SN (Ferrari et al., 2006; Pott Godoy et al., 2010). This conclusion is supported by experiments performed in two different animal models. In one, IL-1β expression in the SN was achieved via adenoviral vector inoculation in that region (Ferrari et al., 2006). On the other hand, the property of adenoviral vectors to retrogradely deliver transgenes from the axonal terminals to the cell bodies was used (Pott Godoy et al., 2010). In both models, IL-1β-mediated neurodegeneration and motor symptoms were detected 21 days after adenoviral administration although IL-1β expression was detected much earlier (7 days). Therefore, sustained IL-1β expression was required for delayed neurodegeneration and motor symptoms to occur. Highlighting the relevance of a sustained in contrast to the acute expression of IL-1β, the acute injection of IL-1β in the SN was not toxic for dopaminergic neurons if the cytokine was injected alone (10 ng or 1000 U) or in combination with 1000 U of TNF-α and 100 U of interferon-gamma (IFN-*γ*; Castaño et al., 2002; Depino et al., 2003). A slightly different result was reported by other group where the acute injection of a similar dose of IL-1β in the medial forebrain bundle produced a 12.2% decrease in the cell counts in the SN 14 days post administration, but this decrease was not statistically different from the cell counts of control animals (Carvey et al., 2005a). Moreover, IL-1β induced by the acute administration of lipopolysaccharide (LPS) in the SN was not toxic (Koprich et al., 2008; Pott Godoy et al., 2008). Over all, the available data suggest that sustained but not acute IL-1β expression at pro-inflammatory levels, has univocal toxic effects in the SN.

Concerning the state in which a dopaminergic neuron perceives IL-1β signaling, it is important to notice that during neuronal cell loss in PD, and also in prion and AD models, the surrounding microglial cells are activated to a particular state defined as “primed” or “atypical” (Perry et al., 2002; Depino et al., 2003; Cunningham et al., 2005; Pott Godoy et al., 2008). In this state of activation, microglial cells change their morphology, possess phagocytic capacity but their array of secreted molecules do not provoke a pro-inflammatory environment, as previously assumed. In particular in PD, IL-1β mRNA levels are increased in the degenerating SN after 6-OHDA administration in the striatum but this increment is not reflected at the protein level (Depino et al., 2003). In other words, the activation of microglial cells during 6-OHDA-mediated neurodegeneration augments IL-1β transcription but not translation, inhibiting the assumed pro-inflammatory effect of IL-1β production at the protein level. However, the transcriptional block of IL-1β protein expression during neurodegeneration can be released by the administration of a sub-toxic dose of an inflammogen such as LPS in the degenerating SN at the time when microglial cells are already “primed” (Pott Godoy et al., 2008). This treatment caused increased IL-1β production, which in turn exacerbated neurodegeneration in the SN and produced earlier and increased motor symptoms in the treated animals (Pott Godoy et al., 2008). The blockade of IL-1β biological activity inhibits the observed exacerbation, implicating this cytokine as a main mediator of the observed effects. In conclusion, a central sub-toxic dose of LPS that does not produce neuronal cell death *per se* in the SN can exacerbate on-going neurodegeneration via IL-1β production from primed microglial cells.

Importantly, in clinical terms, nigral neurodegeneration elicited by two different treatments can be exacerbated from the periphery by eliciting a sustained, succeeding intravenous inflammatory stimulus (Pott Godoy et al., 2008, 2010). Interestingly, in both animal models tested, no neuronal death was observed if the systemic inflammation was elicited in control animals suffering no previous neurodegeneration (Pott Godoy et al., 2008, 2010).

In turn, Koprich et al. (2008), studied the effects of a pro-inflammatory stimulus before instead of after 6-OHDA administration. They have shown that one stimulus of LPS in the SN capable of inducing IL-1 production is not sufficient to produce tyrosine hydroxylase (TH) neuronal loss *per se*, but in the presence of a second challenge with 6-OHDA in the striatum, the death of TH neurons was higher in the animals which received the pro-inflammatory and neurodegenerative stimuli (Koprich et al., 2008). On the other hand, demonstrating the importance of concentration, timing, and nature of the inflammatory insult studied, Saura et al. (2003) have shown that an acute infusion of a high dose of recombinant IL-1β (20 ng) in the SN of rats 5 days prior to the injection of 6-OHDA in the striatal region, protects dopaminergic cellular bodies from 6-OHDA inducing a marked astroglial but no microglia activation and preventing motor dysfunctions. Therefore, even though most of the available evidence suggests that an inflammatory stimulus previous to a neurodegenerative treatment exacerbates neuronal cell death, protective effects under specific circumstances cannot be ruled out.

Several lines of evidence suggest that astrocytes upregulate the expression of inflammatory cytokines such as IL-1α, IL-1β, and IL-6 and the release of TNF-α and IL-6 under pathological conditions (Lee et al., 2010; Rappold and Tieu, 2010). In addition to directly release pro-inflammatory cytokines, astrocytes can also be activated by cytokines such as TNF-α and IL-1β from microglia, leading to production of reactive oxygen and nitrogen species. For example, a recent study demonstrated in a co-culture model, that astrocytes enhance microglial inflammatory responses through an NF-κB-dependent mechanism, leading to more dopaminergic toxicity (Saijo et al., 2009); for a more in-depth review on the functional role of astrocytes in PD, please refer to Rappold and Tieu (2010).

## TNF-α: GENERALITIES

The existence of an anti-tumoral response of the immune system has been described about 100 years ago (Tsung and Norton, 2006). In 1968, TNF-α was identified as a soluble cytokine able to exert significant cytotoxicity on many tumor cell lines and to cause tumor necrosis in certain animal model systems (Kolb and Granger, 1968). The cDNA of TNF-α was cloned and several years later, two membrane receptors were identified (Old, 1985). TNF-α is the prototypic member of a large cytokine family, the TNF ligand family. TNF-α is primarily produced as a type II transmembrane protein arranged in stable homotrimers. From this membrane-integrated form the soluble homotrimeric cytokine [(soluble TNF-α (sTNF-α)] is released via proteolytic cleavage by the metalloprotease TNF-α converting enzyme (TACE). The soluble 51-kDa trimerics TNF tends to dissociate at concentrations below the nanomolar range, thereby losing its bioactivity. The members of the TNF-α ligand family exert their biological functions via interaction with their cognate membrane receptors, comprising the TNF receptor (TNFR) family (McCoy and Tansey, 2008).

Two receptors, TNFR1 (TNF receptor type 1; CD120a; p55/60) and TNFR2 (TNF receptor type 2; CD120b; p75/80) bind membrane-integrated TNF (memTNF) as well as sTNF (Banner et al., 1993; McCoy and Tansey, 2008). TNFR1 is constitutively expressed in most tissues, whereas expression of TNFR2 is highly regulated and is typically found in cells of the immune system. Generally, the importance of TNFR2 is likely to be underestimated, because this receptor can only be fully activated by memTNF, but not sTNF (McCoy and Tansey, 2008; Naude et al., 2011). The cause for this difference is not fully understood yet, but the different stabilities, that is half-lives, of the individual ligand/receptor complexes may contribute to this (Naude et al., 2011). The extracellular domains of both receptors can be proteolytically cleaved, yielding soluble receptor fragments with higher potential neutralizing capacity compared to their membrane-integrated forms (Wallach et al., 1991). TNF-α neutralizing agents for clinical use that were constructed on the basis of the soluble receptors have therefore been engineered as dimeric immunoglobulin G (IgG) fusion proteins. Like TNF, TNFR2 is cleaved by TACE. The processing enzyme(s) responsible for TNFR1 cleavage is still undefined, but TNFR1 cleavage is obviously an important step in the regulation of cellular TNF-α responsiveness, as cleavage-resistant TNFR1 mutations are linked with dominantly inherited autoinflammatory syndromes (TNFR1-associated periodic syndromes, TRAPS). The intracellular domains of TNFR1 and TNFR2 that do not possess any enzymatic activity define them as representatives of the two main subgroups of the TNFR family, the death domain (DD)-containing receptors and the TNF receptor-associated factor (TRAF)-interacting receptors, respectively. TNFR1 contains a protein–protein interaction domain, called DD. The DD can recruit other DD-containing proteins and couples the death receptors to caspase activation and apoptosis. In addition, TNFR1 is also a potent activator of gene expression via indirect recruitment of members of the TRAF family, TRAF2, 5, and 6 (Yoon et al., 2008). TNFR2 directly recruits TRAF2, induces gene expression and intensively crosstalk with TNFR1. Identically as IL-1β signaling pathway, members of the TRAF family activate nuclear factor NF-κB which induces gene expression (Wong et al., 1998). Activation of IL-1β, IL-6, and TNF-α pathways seems to have a negative regulation loop by microRNA miR-146, which has IRAK1 and TRAF6 as targets (Taganov et al., 2006) reviewed in Boldin and Baltimore (2012).

Of interest, TNFRs can have counteracting functions, at least in neuronal tissues, as recently demonstrated in a murine model of retinal ischemia, where TNFR1 apparently aggravated tissue destruction, whereas TNFR2 was protective via activation of PKB/Akt. It has been proposed that TNFR1 or 2 specific agonists and antagonists could mediate specific protective effects in PD (McCoy and Tansey, 2008). In other words, it was proposed that memTNF which is crucial in maintaining a normal innate immune response to infections (Fremond et al., 2005; Saunders et al., 2005; Torres et al., 2005; Alexopoulou et al., 2006; Zalevsky et al., 2007; Allenbach et al., 2008; Naude et al., 2011) should remain active, while sTNF should be pharmacologically inhibited.

## FUNCTIONAL ROLE OF TNF-α IN PARKINSON’S DISEASE

During chronic or acute neuroinflammation high levels of sTNF are found in the cerebrospinal fluid and post-mortem brains of PD patients as well as in animals treated with dopaminergic neurotoxins as MPTP and 6-OHDA used to model nigral degeneration in non-human primates and rodents (Boka et al., 1994; Hunot et al., 1999; Mogi et al., 2000; Sriram et al., 2002; Barcia et al., 2005; Nagatsu and Sawada, 2005). High expression of TNF at the site where neurological damage occurs, suggests that this potent pro-inflammatory cytokine is a mediator of neuronal injury, and therefore, in principle, a feasible target for the treatment of PD.

Many genetically engineered mouse models were designed in order to elucidate the contribution of TNF-α in neurodegeneration. It should be noticed that this models have limitations and they may not exactly reflect what happens in the brain of PD patients. Despite of these restrictions these models had proven to be very useful unraveling the role of TNF-α in this disease.

The effects of TNF-α on the nigrostriatal pathway were investigated by administration of MPTP in knock-out mice lacking TNF-α, or TNFRs. First reports showed that TNF-α was upregulated in the striatum after MPTP administration (Sriram et al., 2002; Ferger et al., 2004). Null mice for the TNF-α gene showed diminished dopamine content loss in the striatum after MPTP administration but no differences in TH-positive cells in the SN, suggesting an overall detrimental effect of TNF-α on dopamine metabolism (Ferger et al., 2004). However, the same group reported that neither TNFR1 nor TNFR2 gene ablation showed protection against MPTP chronic neurotoxicity, suggesting a TNFR-independent mechanism (Leng et al., 2005). Additional work showed that double knock-out mice for both TNFR were protected against the effect of MPTP (Sriram et al., 2002). On the contrary, Rousselet et al., 2002 did not find differences in the number of nigral dopaminergic neurons between the wild type mice and the same double TNFR knock-out mice after MPTP administration. In this last work, the striatal dopamine level was even slightly decreased in the knock-out mice, indicating a potential beneficial effect of TNF-α.

These conflicting results can be attributed to several methodological issues. The doses of MPTP used in these studies were different [15 mg/kg on 8 consecutive days in Leng et al. (2005), 12.5 mg/kg in Sriram et al. (2002), and 60 mg/kg in Rousselet et al. (2002)]. Also, Leng et al. (2005) performed their analysis at day 15 (7 days after the last injection) while Sriram et al. (2002) focused their analysis between 24 and 48 h after MPTP injection and Rousselet et al. (2002) did it at day 10 p.i. These issues can account for the dissimilar results since different regimes of administration of MPTP lead to dramatic changes in the mechanism of cell death (Furuya et al., 2004). Despite this fact, it is still unclear whether TNF-α possess neurotoxic properties and by which TNFR may exert its action in the MPTP model.

Similar contradictory results have been reported in rats injected with the neurotoxin 6-OHDA in the striatum. McCoy et al. (2006) reported that the toxic effects of 6-OHDA could be ameliorated by a synthetic dominant negative TNF-α inhibitor called XENP345 only when infused in the SN, but had no effects in the striatum (McCoy et al., 2006). Furthermore, inhibition of sTNF by injection of a lentiviral vector encoding a dominant negative TNF-α in the SN of rats prevented further loss of nigral DA neurons (McCoy et al., 2006; Harms et al., 2011). On the contrary, Gemma et al. (2007) found that early inhibition of TNF-α by an antisense oligodeoxyribonucleotide (days 1–7 after 6-OHDA insult) could be neurotoxic, but late inhibition (days 7–15) was neuroprotective (Gemma et al., 2007). These finding could indicate a dual role of TNF-α, being neuroprotective during the early steps of injury and neurotoxic when chronically induced.

*In vitro*, TNF-α administration or expression has been shown to be toxic to dopaminergic neurons (McGuire et al., 2001; Gayle et al., 2002; Sriram et al., 2002, 2006). *In vivo*, many reports show detrimental effects of TNF-α injection or overexpression in the SN, but contrary results have also been reported.

Following acute TNF-α administration (1000 U or 20 ng) in the SN, Castaño et al. (2002) could not detect a degenerative effect. In line with this observation, the overexpression of TNF-α via adenoviral vectors in the SN (eliciting an average of 500 pg of TNF-α) did not evoke a toxic effect on dopaminergic neurons up to 7 days after beginning of its expression (De Lella Ezcurra et al., 2010). However, in one report the acute administration of a 100- to 400-fold higher dose of TNF-α than in the previous study triggered dopaminergic cell loss in the SN at 14 but not 7 days post-inoculation (Carvey et al., 2005b). Therefore, in conclusion, most of the available evidence suggests that acute TNF expression at pathophysiological levels is not overtly toxic in the SN.

In experiments where TNF-α was expressed chronically, toxic effects of TNF-α were clearly observed. An early report by Aloe and Fiore (1997) showed that TNF-α overexpression in the CNS of transgenic mice lead to reduced TH immunoreactivity in the striatum. The effects of chronic, upregulated low levels of TNF-α were investigated by De Lella Ezcurra et al. (2010). Intranigral injection of an adenoviral vector encoding soluble mouse TNF-α providing chronic expression of this cytokine in rats showed a progressive neurodegenerative effect which causes forelimb akinesia and a distinct inflammatory response in the rat brain (De Lella Ezcurra et al., 2010). These effects were observed from day 14 post adenoviral inoculation. In addition, Chertoff et al. (2011) studied the effect of TNF-α in the SN of adult mice inducing the chronic expression of this cytokine using a combination of hypomorphic mice where TNF-α expression was controlled by the endogenous engrailed promoter, adenoviral vectors, and the CRE/loxP system. In line with the finding discussed before, chronic upregulated expression of TNF resulted in a progressive loss of DA neurons in the SN and their terminals and monocyte/macrophage recruitment (Chertoff et al., 2011). All together these results indicate that long term expression of pro-inflammatory levels of TNF-α [or acute but very high expression,as reported by Carvey et al. (2005b)] seems to be necessary to exert univocal and toxic effects in the SN.

However, if lower levels of TNF-α were expressed in the SN, a transient neuroprotective effect against 6-OHDA toxicity in the SN and in the striatum was observed (Chertoff et al., 2011). In developmental studies, if the TNF-α expression was elicited during the second week of pregnancy by LPS injection, a toxic effect on adult dopaminergic neurons was detected in the adult naïve or 6-OHDA challenged SN (Ling et al., 2002, 2004; Carvey et al., 2003).

The cholinergic anti-inflammatory pathway should also be taken into account in order to understand the contribution of TNF-α in PD. This pathway regulates the innate immune response after injury or infection. After an insult, TNF-α (and other cytokines) are produced by the cells of the innate immune system, augmenting and prolonging the inflammatory response by activating other cells to release many cytokines (i.e., IL-1β). This initial beneficial response can be detrimental if it lasts for a long time or it is exacerbated. As a way to control this response, TNF-α stimulates the afferent branch of the vagus nerve which conveys this signal to the CNS. In turn, the motor branch of the vagus nerve releases acetylcholine in the periphery. In particular, this neurotransmitter interacts with the alpha7 subunit of the nicotinic acetylcholine receptor expressed in macrophages, and other cytokine-releasing cells. This interaction activates intracellular signal transduction which inhibits release of pro-inflammatory cytokines, in particular TNF-α. This negative feedback circuit regulates the inflammatory response, maintaining homeostasis (Tracey, 2002), reviewed in Tracey et al. (2008). A similar cholinergic pathway exists in the brain, regulating microglial activation (Shytle et al., 2004). In addition, IL-1β promotes the synthesis of acetylcholinesterase (Li et al., 2000), which would decrease acetylcholine and promote inflammation.

These findings may have therapeutic consequences. Vagal stimulation has proven effective for the treatment of epileptic seizures refractory to pharmacological treatments in adult patients (Ben-Menachem et al., 1994; Groves and Brown, 2005; Tecoma and Iragui, 2006) and possible applications for PD patients are foreseen (Bokkala-Pinninti et al., 2008). Specifically in PD, an inverse correlation has been found between nicotine (a ligand for the alpha7 nicotinic receptor) consumption and the risk of PD development. Regarding this, Park et al. (2007) reported that micromolar concentration of this compound significantly reduced release of TNF-α and loss of DA neurons* in vitro*, in a LPS model.

## INTERACTIONS BETWEEN IL-1β AND TNF-α

The neuroimmunology of the brain parenchyma differs in many aspects from the immune response elicit systemically (for extensive review see Lowenstein and Castro, 2003; Perry et al., 2010; Roca et al., 2011). In relation with the interaction between IL-1β and TNF-α, the cytokine network in the brain parenchyma differs from the periphery. Whereas in the periphery IL-1β production leads to TNF production (and vice versa) in an overwhelming majority of models and experimental conditions (Luger and Schwarz, 1990; Creasey et al., 1991; Chuluyan et al., 1998), the mutual induction of one cytokine by the other is restricted in the CNS. It has been demonstrated that the injection in the brain parenchyma of IL-1β triggers IL-1β mRNA and protein synthesis but not TNF-α expression (Blond et al., 2002; Depino et al., 2005). In addition, TNF-α injection in the brain parenchyma does not induce TNF-α itself or any IL-1β beta expression (Blond et al., 2002). These observations hold true even if IL-1β or TNF-α are expressed chronically in the SN or the striatum using adenoviral vectors (Ferrari et al., 2004, 2006; De Lella Ezcurra et al., 2010).

In parallel and in accordance to a restricted presence of one cytokine and not the other in the brain parenchyma, the pattern of leukocyte recruitment to the brain triggered by each of these two primordial pro-inflammatory cytokines is different. IL-1β seems to recruit preferentially neutrophils and polymorphonuclear cells; while TNF-α calls for mononuclear cell infiltration (Anthony et al., 1997; Schnell et al., 1999; Blond et al., 2002; Ferrari et al., 2004, 2006; Depino et al., 2005; De Lella Ezcurra et al., 2010).

Therefore, under the light of the restricted induction of each other, the evidence described here on their functional role in PD could be mainly associated to one cytokine and not to the other.

Given the fact that around 30% of PD patients may develop symptoms related to AD, it is relevant to note that the current evidence points also compellingly toward a central role for inflammation in AD. The observed neuroinflammation would create a chronic and self-sustaining interaction between activated microglia and astrocytes, stressed neurons, and Aβ plaques (Rubio-Perez and Morillas-Ruiz, 2012). In addition, a clinical study involving 300 patients correlates cognitive decline with the occurrence of systemic inflammation in AD patients (Holmes et al., 2003). Overexpression of mutant forms of amyloid β-protein precursor (APP) in the brains of transgenic mice produced amyloid plaques surrounded by activated microglia and reactive astrocytes and upregulated IL-1β, IL-6, and TNF-α, which resembled the alterations found in patients with AD (Morgan et al., 2005; Jin et al., 2008). In this sense, therapies using an anti-TNF-α fusion protein produced sustained clinical improvement in a 6-month, open-label pilot study in patients with AD ranging from mild to severe (Tobinick, 2009).

## CONCLUSION

**FIGURE 1:**
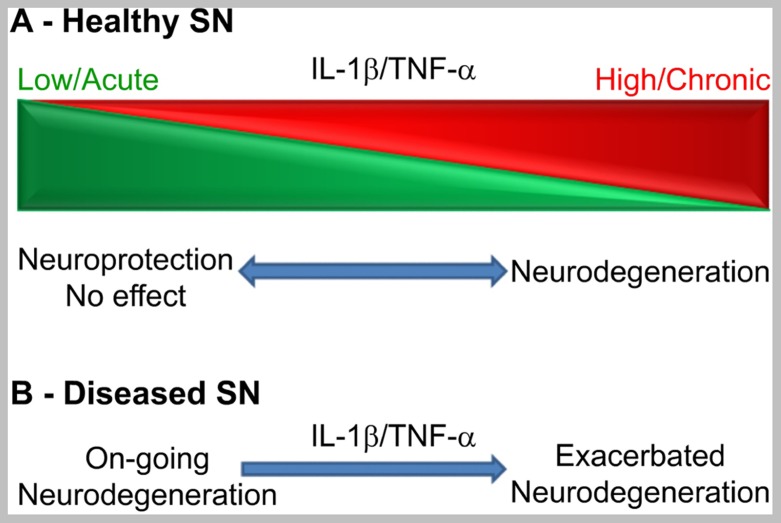
**Schematic representation of a working framework to study the functional effects of IL-1β and TNF-α on dopaminergic neurons *in vivo* according to duration and levels of expression in the healthy SN (A) or in the degenerating SN (B).** Clear effects are only seen at the extremes of the scheme or when a pro-inflammatory stimulus hits the already degenerating SN.

In conclusion, as expected for molecules with pleiotropic functions, the net, univocal effects of IL-1β or TNF-α on dopaminergic neuronal viability *in vivo* depends on a number of variables. Although cytokine biology is not prone to generalizations, in this case the available evidence tempts us to suggest that, in the healthy SN, these variables can be mainly circumscribed to duration and levels of expression (see **Figure 1A**). As stated above, IL-1β and TNF-α effects in the brain could be distinctly separated from each other, since they are not mutually inducible in the brain parenchyma. However, the influence of these variables on their net functional effect seems to be similar in general terms. In the healthy SN (**Figure 1A**), the acute administration or expression of both cytokines seems to have no dramatic effect on dopaminergic neurons in the SN, unless expressed at supra-physiological levels (Castaño et al., 2002; Depino et al., 2003; Saura et al., 2003; Carvey et al., 2005a; Koprich et al., 2008). On the contrary, sustained levels of IL-1β or TNF-α have been associated with neurodegeneration in the SN (Aloe and Fiore, 1997; Ferrari et al., 2006; De Lella Ezcurra et al., 2010; Pott Godoy et al., 2010; Chertoff et al., 2011).In terms of dosage, low levels of these cytokines have produced either no or neuroprotective effects (Depino et al., 2003; Chertoff et al., 2011) whereas high, pro-inflammatory levels produced univocally neuronal demise in the naïve SN (Ferrari et al., 2006; Pott Godoy et al., 2010; Chertoff et al., 2011). The overwhelming evidence accumulated suggests that the combination of both variables increased the prediction of the net biological effect of IL-1β or TNF-α on the SN (**Figure 1A**). In other words, sustained and high IL-1β and TNF-α expression will univocally lead to neurodegeneration, while acute and low expression will lead to no or neuroprotective effects.

It is also clear that, in the degenerating SN, when microglial cells are primed, acute and sub-toxic pro-inflammatory stimuli irreversibly leads to increased neurodegeneration (**Figure 1B**; Pott Godoy et al., 2008, 2010). If IL-1β is expressed before neurodegeneration, it can increase susceptibility to 6-OHDA (Koprich et al., 2008) or have neuroprotective effects (Saura et al., 2003) depending on other variables. In the case of TNF-α, the only report studying the effects of low TNF-α levels in the adult SN before 6-OHDA administration shows a neuroprotective effect (Chertoff et al., 2011). However, several developmental studies have shown a toxic effect on adult dopaminergic neurons of LPS-derived TNF-α when administered during the second gestation week in the rat (Ling et al., 2002, 2004; Carvey et al., 2003). Therefore, when the SN is subjected to IL-1β or TNF-α before a neurodegenerative stimulus, the final effect depends on other variables.

Hence, if these variables are affecting IL-1β and TNF-α effects on dopaminergic viability, how can this information be translated to future treatments to PD patients where these variables are uncontrollable and not measurable?

Indeed, a similar scenario has occurred in the MS field. MS is an autoimmune demyelinating disease whose inflammatory component is much more characterized and typical than in PD. In models of MS, TNF-α accelerated the onset of disease and increased apoptosis of oligodendrocytes (Caminero et al., 2011). Based on numerous and sound studies *in vitro* and *in vivo* demonstrating toxic effects of TNF-α, clinical trials using anti-TNF-α molecules were approved and started (Caminero et al., 2011). Unfortunately, anti-TNF-α therapies failed to improve, or even worsened, the symptoms of MS patients treated (Caminero et al., 2011). Later it was found that TNF-α can have also beneficial effects on MS progression such as promoting the regression of myelin-specific T cell activity at later time points (Kassiotis et al., 2001) and inducing the proliferation of oligodendrocyte progenitors (Arnett et al., 2001). Also, TNFR1 seems to mediate demyelination while TNFR2 preferentially induces remyelination, calling for TNFR-specific therapies (Caminero et al., 2011). Therefore, it can be suggested that the disappointing results of the anti-TNF-α clinical trials against MS were due to the inhibition of previously unrecognized beneficial effects of this cytokine and/or unspecific receptor activation.

Thus, how can we learn from the MS field and design clinical trials with a higher probability of achieving therapeutic efficacy?

Based on the evidence discuss in this review, we are certain that general anti-IL-1β or TNF-α therapies against PD have low probability of success. In a similar way, general anti-inflammatory treatments may inhibit toxic but also beneficial effects of TNF-α, IL-1β, or other cytokines.

One possibility to increase the success rate of future clinical trials could be to use receptor-specific agonists or antagonists to circumvent undesired effects of general anti-inflammatory or specific anti-IL-1β or TNF-α therapies against PD. This option has been suggested especially for TNF-α, where the different biological effects of TNFR1 and TNFR2 have been more exhaustively studied (McCoy and Tansey, 2008). In addition, taking advantage of the biological differences of memTNF and sTNF on their receptor binding, several specific inhibitors have been designed on these bases (Moreland, 1998; Deswal et al., 1999; Zalevsky et al., 2007; Gonzales et al., 2008; Mukai et al., 2009).

Finally, we believe that there are common and specific downstream mediators of the toxic effects of IL-1β and TNF on dopaminergic neurons in the SN. The identification of these IL-1β- or TNF-derived molecules with univocal toxic effects should open the possibility to inhibit IL-1β or TNF-α-detrimental effects while sparing their neuroprotective properties, increasing the probability of efficacy in a clinical setting. A similar, but reverse rational can be applied to neuroprotective-only molecules. Fortunately, numerous animal models and experimental set-ups have been generated in the past 10 years to be able to identify these candidate molecules.

Undoubtedly, neuroinflammation is no longer regarded as the expected consequence of neuronal demise leading to the clearance of cell debris, but a phenomenon dramatically affecting PD progression and therefore a target for neuroprotective or disease-modifying therapies. The actual challenge is to reach the clinic with compounds univocally targeting the detrimental effects of neuroinflammation, sparing possible beneficial effects of these pleiotropic molecules.

## Conflict of Interest Statement

The authors declare that the research was conducted in the absence of any commercial or financial relationships that could be construed as a potential conflict of interest.
